# Smoking prevalence and smoking cessation services for pregnant women in Scotland

**DOI:** 10.1186/1747-597X-5-1

**Published:** 2010-01-21

**Authors:** David M Tappin, Susan MacAskill, Linda Bauld, Douglas Eadie, Debbie Shipton, Linsey Galbraith

**Affiliations:** 1 Paediatric Epidemiology and Community Health Unit, Child Health Section, Division of Developmental Medicine, University of Glasgow, Glasgow, G3 8SJ, UK; 2 Centre for Tobacco Control Research, Institute for Social Marketing, University of Stirling and Open University, Stirling, FK9 4LA, UK; 3 UK Centre for Tobacco Control Studies, Dept of Social and Policy Sciences University of Bath, Bath BA2 7AY, UK; 4 Information Services Division, NHS National Services Scotland, Gyle Square, 1 South Gyle Crescent, Edinburgh EH12 9EB, UK

## Abstract

**Background:**

Over 20% of women smoke throughout pregnancy despite the known risks to mother and child. Engagement in face-to-face support is a good measure of service reach. The Scottish Government has set a target that by 2010 8% of smokers will have quit via NHS cessation services. At present less than 4% stop during pregnancy. We aimed to establish a denominator for pregnant smokers in Scotland and describe the proportion who are referred to specialist services, engage in one-to-one counselling, set a quit date and quit 4 weeks later.

**Methods:**

This was a descriptive epidemiological study using routinely collected data supplemented by questionnaire information from specialist pregnancy cessation services.

**Results:**

13266 of 52370 (25%) pregnant women reported being current smokers at maternity booking and 3133/13266 (24%) were referred to specialist cessation services in 2005/6. Two main types of specialist smoking cessation support for pregnant women were in place in Scotland. The first involved identification using self-report and carbon monoxide breath test for all pregnant women with routine referral (1936/3352, 58% referred) to clinic based support (386, 11.5% engaged). 370 (11%) women set a quit date and 116 (3.5%) had quit 4 weeks later. The second involved identification by self report and referral of women who wanted help (1195/2776, 43% referred) for home based support (377/1954, 19% engaged). 409(15%) smokers set a quit date and 119 (4.3%) had quit 4 weeks later. Cost of home-based support was greater. In Scotland only 265/8062 (3.2%) pregnant smokers identified at maternity booking, living in areas with recognised specialist or good generic services, quit smoking during 2006.

**Conclusions:**

In Scotland, a small proportion of pregnant smokers are supported to stop. Poor outcomes are a product of current limitations to each step of service provision - identification, referral, engagement and treatment. Many smokers are not asked about smoking at maternity booking or provide false information. Carbon monoxide breath testing can bypass this difficulty. Identified smokers may not be referred but an opt-out referral policy can remove this barrier. Engagement at home allowed a greater proportion to set a quit date and quit, but costs were higher.

## Background

Although the risks of smoking during pregnancy for both mother and child are well established, [1] smoking throughout pregnancy is still common with reported smoking rates varying from 21% in Scotland [2] to 17% in England [3]. Smoking prevalence increases with deprivation and this is certainly true of Scotland, where in 2008 30% of pregnant women in the most deprived areas self-reported as current smokers compared to 7% in the least deprived areas [2].

Scotland has national targets to reduce the proportion of women who smoke during pregnancy (from 29% in 1995 to 20% by 2010), and to reduce inequalities, increasing the rate of improvement in the most deprived communities by 15% [4]. NHS Stop Smoking Services have an important role to play in achieving these targets. Recommendations for the provision of smoking cessation support to pregnant women were made in the Smoking Cessation Guidelines for Scotland [5]. Health boards have sought to build on these guidelines by establishing tailored specialist services for pregnant women. Some services are now well established, while others are at an earlier stage of development.

In order to develop a coherent service, good information is needed about engagement of pregnant smokers with specialist cessation services and the success of their treatment in terms of biochemically validated success at quitting.

The process of supporting women to quit can be divided into five stages. Stage 1 identifies all smokers preferably before pregnancy, but definitely early in pregnancy to establish a denominator. Usually all women in Scotland are asked by their booking midwife if they are a *current, former, or never *smoker. This data is returned to the Information Services Division of NHS National Services Scotland on the Scottish Morbidity Record (SMR02) from each maternity hospital. If the data is not available either because the women were not asked or because the answer was not recorded the smoking status is shown as *not known*. This information is not confirmed by routinely testing for breath carbon monoxide or serum cotinine.

Stage 2 involves referral by the midwife after the maternity booking visit to specialist smoking cessation services. Usually brief intervention is provided by the booking midwife who asks the client if they would like further help via referral to specialist smoking cessation services. Those who agree to referral 'opt-in' to the smoking cessation services. Generic (i.e. for the general population, not specifically for pregnant women) smoking cessation services have been in place in Scotland since 2000 but few pregnant smokers were referred or attended. More recently specialist smoking cessation services have been established for pregnant women in some areas and not others. Health Boards are provided with funding for smoking cessation but to an extent they can decide the way they want to target that resource. Some Health Boards have developed specialist smoking cessation services for pregnant women. Others spent their smoking cessation funding allocation in a different way. Findings from local studies suggest that referrals have increased with the development of specialist services for pregnant smokers[6].

Stage 3 describes the reach of services and is termed 'engagement' - defined as having at least one face-to-face therapeutic encounter with a person who is providing specialist smoking cessation support. This face-to-face encounter is usually provided in either the home or at a special clinic visit.

Stage 4 is setting a quit date.

Stage 5 is quitting 4 weeks after the quit date which should be biochemically verified.

Stages 4 and 5 information is collected by all NHS smoking cessation services in Scotland as part of the agreed National Minimum Dataset (MDS), [7] and national monitoring of cessation services and is returned to the Information Services Division, NHS National Services Scotland.

This paper describes the available information for each of these stages in Scotland by maternity unit and by area where established specialist smoking cessation services for pregnant women are in place. The routinely collected data has been augmented by questionnaire data collected as part of a mapping project to describe pregnancy smoking cessation services in Scotland funded by NHS Health Scotland [8].

## Methods

This observational study employed mixed methods to describe the population of pregnant smokers in Scotland during 2005 and examine rates of referral, engagement, and quit attempts including short-term quit rates for women giving birth in 2006.

The denominator of self-reported smoking at maternity booking is gathered routinely as part of the maternity data collection system which is returned in the Scottish Morbidity Reporting system (SMR02) on an annual basis to the Information Services Division, NHS National Services Scotland. Maternity care is orientated around maternity hospitals and all women who book for maternity care have an SMR02 return. Some women either deliver away from their booking hospital or do not attend for antenatal care and arrive at maternity hospitals in labour. The data for the year 1st April 2004 to 31st March 2005 was used so that corrections could be made for births in each maternity unit as 2005 was the latest revised data available for number of births in each hospital [9]. Rates of referral, engagement, and quit attempts including short-term quit rates were gathered by questionnaire from individual services [8] supplemented by data from the National Minimum Dataset (MDS) [7] for the period 1st March 2005 to 28 February 2006.

Ethics enquiry by NHS Health Scotland confirmed that this project was service evaluation and did not require to be reviewed by an ethics committee.

### Stage 1 - Establishing the denominator of pregnant smokers in Scotland

Routine smoking prevalence data [9] captured at maternity booking (8-12 weeks gestation) via the Scottish Morbidity Record (SMR02) held at the Information and Statistics Division (ISD) NHS National Services Scotland was examined in detail by DS. Table 1 illustrates that different approaches can be taken to interpret SMR02 data, described here as comprehensive or pragmatic. A simple, or 'pragmatic', method of identifying smokers using the SMR02 flat file was conducted under the direction of ISD staff, which extracted smoking data from the maternity booking appointment only. This was compared with a more extensive ('comprehensive') method; involving the extraction of all possibly conflicting smoking data recorded in the SMR02 from any one pregnancy. For example at subsequent antenatal visits, for premature labour or pre-eclampsia, smoking data is usually collected. We concluded that the pragmatic approach provided an adequate estimate of information available and it is the basis for our analyses. In table 1 the pragmatic analysis reveals that 22.1% of pregnant women in 2005 were identified as current smokers, with 63.3% recorded as never smokers, 8.7% as former smokers and 5.9% of cases with smoking status unknown.

**Table 1:** Smoking at maternity booking for women delivering in 2005 by data extraction method from SMR02 flat file held by Information Services Division (ISD) Scotland

	Smoking Status in 2005								
	**unknown**		**former**		**current**		**never**		**Total**

**Approach**	**n**	**%**	**n**	**%**	**n**	**%**	**n**	**%**	

Comprehensive	2710	5.5	4369	8.8	11317	22.9	31112	62.8	49508§

Pragmatic	2913	5.9	4345	8.7	10990	22.1	31529	63.3	49777§

§ A simple or 'pragmatic' analysis of the SMR02 flat file was conducted under the direction of ISD staff. This was compared with a more extensive ('comprehensive') trawling of that file, which includes multiple entries for maternal smoking on a few women admitted to maternity units for antenatal care (e.g. due to premature labour or preeclampsia). We concluded that the pragmatic approach provided an adequate estimate of information available.

Difference between records obtained using pragmatic and comprehensive approach accounted for by:

•Duplicate records: 94 women with duplicate records counted only once in Comprehensive approach.

•Missing admission year: 175 records with missing admission year were excluded from Comprehensive approach.

It is clear that SMR02 does not capture all women who give birth with information at maternity booking. Total births in Scotland for year ending 31st March 2005 was 52721 - Information Services Division and 53849 - General Register Office.

A number of problems were noted when reviewing routine data on smoking in pregnancy and the SMR02. These include: under reporting, recording problems, and problems with data from particular hospitals.

**1. Maternal under-reporting**: Not all women will admit that they are smokers at maternity booking. This has been found in the UK and internationally. In New Zealand, for example, 20% of smokers mis-reported themselves as non-smokers when asked at maternity booking by their routine midwife, verified by serum cotinine estimation on residual routine pregnancy blood samples in 1994 [10]. In Scotland 17% of smokers defined by cotinine testing misreported themselves at maternity booking as non-smokers [11]. Even if all women were asked about smoking then perhaps 20% of smokers would be missed and not be referred for specialist support.

**2. Recording problems**: The SMR02 data allows us to see that not all women were routinely asked about their smoking status at maternity booking (based on *recording *of whether that question was asked). More than 5% of women in 2005 were recorded as 'not known', meaning no entry was made for smoking on their SMR02 return (Table 1). This problem is distributed unevenly across the maternity units. Most units provided information for more than 97% of SMR02 returns. However, hospitals with high levels of unknown smoking at booking in 2005 included - Perth Royal Infirmary (36% of cases), Princess Royal Maternity (32%), Ninewells (13%) and the Queen Mother's Hospital (8.5%). Most other hospitals had less than 5% 'not known' smoking status[9]. This information can be viewed online by health board [9] for the years 1995 to 2008 and is the measure used for target-setting supported by smoking data collected 10 days after birth at the Health Visitor first visit [9].

**3. Varied levels of returns**: A few hospitals returned SMR02 data very poorly. Among Tayside hospitals, the proportion of births in Ninewells hospital that had an SMR02 return was less than 10%. This resulted from a technical problem with the maternity system used in Ninewells for which a solution was being sought. There were also problems with returns (although less significant) from the Princess Royal Maternity Hospital in Glasgow.

There are a number of potential solutions to these problems with SMR02 data. We have made adjustments which have been agreed with ISD to resolve problems 2 and 3, and to provide an estimate of the true denominator for self-reported smoking. Corrections were made for difference between *Total births *in the hospital in 2005 [9] and *Total booked *in the hospital from the SMR02 2004/5 ISD flat file. Women with *Not known *smoking status ISD flat file were distributed as proportions of *current/former/never *smokers in that hospital - this simple method of replacing unknown data has been backed up by a recent study in the West of Scotland [11].

We have not, however, made any correction for potential under-reporting by women themselves at maternity booking. This means that the figures for the denominator self-reported smokers presented in Table 2 are undoubtedly underestimates of the number of women actually smoking at maternity booking. A study published in the British Medical Journal [11] has shown that 17% of smokers falsely categorise themselves as non-smokers at maternity booking in Scotland. No adjustment has been made to the denominator figure in table 2 to take account of this under-reporting.

**Table 2:** Pregnant smokers in Scotland receiving cessation support during 2005/6 Stage 1 relates to maternity booking from April 2004 to March 2005, Stages 2-5 relate to March 2005 to February 2006 unless stated in the footnote

Health Board and Hospital	***Stage 1 *Self reported current smokers corrected for % unknown and total births in hospital **(% of births)	***Stage 2 *Referred to specialist services **(% self reported smokers)	***Stage 3 *Engaged in face-to- face contact **(% self reported smokers)	***Stage 4 *Women who set a quit date **(% of self reported smokers)	***Stage 5 *Women self-reported quit at 4 weeks post quit date **(% of self reported smokers)	WTE staff providing specialist smoking cessation service* (H - Home C - Clinic)
**Ayrshire and Arran**						
Ayrshire Central	**1100**/3590 (31%)	Generic Services +				None

**Borders**						
Borders General	**292**/1042 (28%)	Generic Services +		4§§	Not Known	None

**Dumfries and Galloway**						
Royal Infirmary	**343**/1305 (26%)	98 (29%)μ	44 (13%)μ	37 (11%)μ	9 (2.6%)μ**	0.5 (H)

**Fife**						
Forth Park	**986**/3324 (30%)	396 (40%) μ	193(20%) μ	102 (10%) μ	39 (4.0%) μ	1.2 (H)

**Forth Valley**						
Stirling Royal Infirmary	**789**/3116 (25%)	New staff appointed Oct'07		Not Known	Not Known	None

**Grampian**						
Aberdeen Maternity	**923**/4183 (22%)	Identified midwives work individual sessions				0.4 +

Elgin	**228**/950 (24%)	None appointed (spring 2007)				

Peterhead	**26**/110 (24%)	None appointed (spring 2007)				

**Greater Glasgow & Clyde**						
Southern General 'breathe'	**664**/3219 (21%)	573 (86%)§	106 (16%)§	93 (14%)§	33 (5.0%)§	0.5 (C)

Princess Royal 'breathe'	**1804**/5570 (32%)	703 (39%)§	146 (8%)§	145 (8%)μ	50 (2.8%)μ	0.5 (C)

Queen Mother's 'breathe'	**884**/3344 (26%)	660 (75%)§	134 (15%)§	132 (15%)§	34 (3.8%)§	0.5 (C)

Vale of Leven CATCH		[159 μ	78 (9%) μ	[50 μ	[12 μ	0.4 (H)
					
Royal Alexandra CATCH	**822**/2710 (30%)	182 (55%)1	Not known	45 (20%)1	24 (4.7%)1	1.2 (H)
					
Greenock services CATCH		115 μ]	Not known	70 μ]	3 μ]	1.0 (H)

**Highlands and Islands**						
Raigmore	**520**/1888 (28%)	Service from Nov'06 (training/cessation support)		Not Known	Not Known	0.5
			
Caithness	**45**/205 (22%)			Not Known	Not Known	

Balfour Hospital, Orkney	**18**/127 (14%)			1§§	1§§	
			
Gilbert Bain, Shetland	**28**/154 (18%)	Generic services +		2§§	1§§	
			
Western Isles	**28**/178 (16%)			Not Known	Not Known	

**Lanarkshire**						
Wishaw General	**1338**/4777 (28%)	Generic Services +		61§§	22§§	None

**Lothian**						
Royal Infirmary	**550**/5792 (9%)	Not Known	Not known	57§§	5§§	2.3

St John's Howden	**625**/2743 (23%)	247 (40%)μ	140 (22%)μ	105 (17%)μ	32 (5.0%)μ	1.0 (H)

**Tayside**						
Ninewells	**1131**/3535 (32%)	Give it up for Baby: first clients April 2007				Community pharmacists

Perth Royal Infirmary	**88**/384 (23%)					None
		Generic Services +				
Montrose Royal Infirmary	**34**/124 (27%)					

**Total for Scotland**	**13266**/52370*** (25%)					

Notes:

Readers will have noticed that Stage 1 refers to smokers identified at maternity booking during the 12 month period April 2004 to March 2005. Stages 2-5 refer to a period March 2005 to February 2006. Little change took place in self-reported current smoking at maternity booking between the year ending March 2005 - 22.5% and the year ending March 2006 21.7% [9]. Much of this difference can be explained by an increase in the 'not known' category from 7.2% to 9.4%.

1. CATCH data [21] from June 2005- May 2006 - Vale of Leven bookers deliver at Queen Mother's and Royal Alexandra, Greenock bookers mostly deliver at Royal Alexandria in Paisley

§ 'breathe' statistics [15] Jan-Dec'06 with booking figures for same period.

The Queen Mother's Hospital delivers patients booked in a geographic area north of the River Clyde where smoking cessation support is provided by the (CATCH) service [21]. It is estimated that 100 smokers from the CATCH service deliver in the Queen Mother's Hospital. Therefore, for clarity, these smokers have been moved from the Queen Mothers Hospital to the Royal Alexandra Hospital so that the separate service models CATCH - home-based and breathe - clinic based can be compared more easily. This was done for the paper describing 'breathe' [15] and it would seem appropriate to repeat this adjustment.

§§ Taken from the National Smoking Cessation Database [West Lothian: St John's Howden, rest of Lothian: Royal Infirmary of Edinburgh]

μ from questionnaire for the mapping exercise

* Active staffing levels may be lower at times, for example absence due to sick leave or difficulty in filling posts.

** 3 months post quit date

*** Total births do not reflect SMR or ISD as births from 2006 used for 'breathe data

+ Generic services are those provided for all smokers and include 'Smokeline' and pharmacy based services not specifically aimed at individual groups such as pregnant smokers.

### Stage 2 - Referral of identified pregnant smokers

Once a pregnant smoker has been identified, they should be offered brief advice to quit by their midwife or GP, and be referred to a smoking cessation specialist [12]. Table 2 (Stage 2) summarises referrals to specialist support services as far as is known and used data from a number of sources described in the notes to the table.

### Stage 3 - Engagement in at least one face to face therapeutic session with a specialist smoking cessation practitioner

Engagement data was collected from individual specialist smoking cessation services identified in the mapping process [8] (Table 2). Some services had not collected this information.

### Stage 4 - Setting a quit date

Once women have engaged with services, an important objective is to encourage them to set a quit date. The quit date is recorded by services and returned to ISD as part of required data for the National Minimum Data Set (MDS) for smoking cessation services in Scotland [7]. This information was not available for all areas, particularly those without specialist smoking cessation services for pregnant women.

### Stage 5 - Short-term 4 week quit rates

Once a quit date has been set, all women are assessed after 4 weeks to see if their quit attempt has been successful, ideally verified by a carbon monoxide breath test. It should be noted that short term quit rates overestimate long term quit rates due to relapse and false reporting especially if biochemical validation is not employed. Even if carbon monoxide testing is employed, abstinence for a few hours allows a light smoker to be falsely verified as quit [13]. Cessation data are now recorded for all smokers who come into contact with NHS smoking cessation services in Scotland (including pregnant smokers) as part of the MDS [7]. For births from March 2005 to February 2006 not all areas were submitting MDS returns, so the data was supplemented by questionnaire data [8] gathered from individual services.

### Specialist smoking cessation practitioner time utilized for this service

These data were made available by individual services during the mapping project [8] which employed a mixed methods approach across four elements, with findings from each element informing those that followed. Element 1 involved telephone enquiries with the main tobacco lead(s) in each health board area to explore service provision (*n *= 16). Element 2 gathered more detailed information about support, incorporating self completion questionnaires for specialist services (*n *= 10) and telephone interviews with senior midwifery staff in the remaining areas (*n *= 10). Element 3 involved site visits to six services in Scotland and England to obtain more detailed insights into service delivery and examples of promising practice (*n *= 28). In parallel with Elements 1 to 3, Element 4 involved an audit of routinely collected data at five different stages of identification and treatment of smokers.

### Other data examined

Carstairs deprivation index [14] based on postcode of residence was collated for pregnant smokers in Glasgow (Table 3) from Stages 1 to 5. Carstairs deprivation category is a small area based system that relates a measure of material deprivation to all residents of a small area - postcode sector based on census data for that sector. It was designed using those census measures strongly correlated with major morbidity and mortality. Carstairs Deprivation category 7 are the most deprived postcode sectors, whereas category 1 are the least deprived. This measure was used by the *breathe *service in Glasgow [8,15] who provided the data for table 3. The reason for including these data is to suggest that material deprivation may not be so important in treating pregnant smokers. A similar proportion of self reported smokers engaged with services from deprived groups and from affluent groups - Table 3). A similar proportion quit smoking from the most affluent groups in Glasgow compared to the most deprived groups. We have no data available from other services outside Glasgow relating to stages 2 to 5.

**Table 3:** Distribution of material deprivation for self-reported pregnant smokers at booking in Glasgow and those who attended specialist pregnancy smoking cessation services from May 2005 to May 2006 (figures slightly different to Table 2 due to time period)

**Carstairs Deprivation Category **[14] *	Stage 1 Self reported smokers	Stage 3 Attended 1st Visit	Stage 4 Set quit date	Stage 5 Successfully quit
	n (column %)	n (column %)	n (column %)	n (column %)
1&2	164 [7]	23 [6]	22 [6]	8 [7]
3-5	773 [31]	99 [27]	91 [27]	35 [31]
6&7	1545 [62]	248 [67]	228 [67]	70 [62]

**Total**	**2842**	**370**	**341**	**113**

* Separation in this way into 3 categories is often performed with categories 1&2 the most affluent, 3-5 middle and 6&7 as people living in the most materially deprived areas.

## Results

### Stage 1

Table 1 shows two different ways to estimate the number of pregnant smokers booking for maternity care. We utilised the pragmatic approach and corrected for differences between the number of SMR02 maternity booking returns and the number of births in each hospital to come to the estimated number of smokers who would self-report their habit in Table 2. We estimate that 13266/52370 (25%) of pregnant women in Scotland self-reported as current smokers at maternity booking in 2005/6. The proportion varied from 32% for the Princess Royal Maternity Hospital in Glasgow (PRMH) and Ninewells hospital in Dundee to less than 9% at the Royal Infirmary in Edinburgh.

### Stage 2

3133/6128 (51%) were referred in areas with specialist smoking cessation services in 2005/6. This ranged from 29% at the Royal Infirmary Dumfries & Galloway to 86% at the Southern General Hospital in Glasgow.

### Stage 3

763/5306 (14%) self reported smokers engaged in face-to-face contact with a specialist smoking cessation practitioner in areas with specialist smoking cessation services in 2005/6 - 22% of self-reported smokers at St John's Hospital West Lothian (StJ), and only 8% at the PRMH, Glasgow.

### Stage 4

779/6128 (13%) set a quit date. This varied from 17% of self-reported smokers at StJ to 8% at PRMH Glasgow.

### Stage 5

Overall in Scotland 236/6128 (3.9%) smokers identified at booking, living in areas with recognised specialist services, self-reported abstinence 4 weeks after their quit date during 2006. About half the quits at 4 weeks were biochemically validated using the carbon monoxide breath test. Self-report quit varied from 5.0% at the Southern General Hospital Glasgow (33/664 - 21 CO validated, 12 no CO validation performed) [15] and St John's Howden West Lothian to 2.6% at the Royal Infirmary Dumfries and Galloway, for example. For areas with clinic-based services with an opt-out policy (Glasgow) - attempt was made by specialist smoking cessation services to phone all identified smokers - 117/3352 (3.5%) of self-reported pregnant smokers quit compared with 119/2776 (4.3%)(z = 1.6, p = 0.1) in areas providing home-based support using an opt-in policy - minimal intervention by routine booking midwife with referral of those who wanted specialist help. For women who set a quit date, 119/409(29%) had quit 4 weeks later with specialist home-based services, 117/370(32%) with clinic-based services and 24/61(35%) with generic services.

### Specialist smoking cessation practitioner time utilized for this service

Five maternity units operated a home-based opt-in service to engage clients with specialist smoking cessation services (Table 2). In these areas 2776(100%) women self reported as smokers at maternity booking, 1197(43%) were referred to specialist smoking cessation services, 570(21%) engaged by having at least 1 face to face contact, 409(15%) set a quit date and 119(4.3%) women quit smoking. The services were staffed by a total of 5.3 whole time equivalent specialist smoking cessation midwives. The PRMH, Queen Mother's and Southern General hospitals in Glasgow operated a clinic-based opt-out service to engage clients in specialist smoking cessation services. In these areas 3352(100%) women self reported as smokers at maternity booking, 1936(58%) smokers were referred to specialist smoking cessation services, 386(12%) engaged, 373(11%) set a quit date and 117(3.5%) women quit smoking. This service was staffed by 1.5 specialist smoking cessation midwives.

### Other data

Table 3 accounts for the self-reported smokers in Glasgow who were served by the three hospitals in the city. It can be seen that the proportion of women living in the most deprived areas (deprivation category 6&7) remains fairly constant, at around two-thirds, from stage 1 (identified smokers) to stage 5 (successfully quit). Overall 386/1938(20%) of women in Glasgow who were referred attended a first clinic visit and therefore engaged with the specialist smoking cessation service. Among those who did not engage: for 733/1938(38%) contact via telephone was impossible due to unobtainable or incorrect number, 549(28%) declined help at telephone contact by specialist services, 273(14%) did not attend the clinic appointment arranged at the first telephone contact [8,15].

## Discussion

### Targets

The Scottish Government has recently set targets that include one for smoking cessation services. This states that "Through smoking cessation services, 8% of your Board's smoking population will be supported to successfully quit (at one month post quit) over the period 2008/9 - 2010/11." It is clear from Table 2 that in no service in Scotland in 2006 did 8% of self-reported smokers quit during pregnancy. The closest achieved was 5.0% by the Southern General Hospital in Glasgow - an opt-out clinic based service - and St John's Howden, West Lothian - an opt-in home-based service. Overall in Scotland only 265/8062(3.2%) smokers living in areas with recognised specialist or good generic services quit smoking during pregnancy in 2006.

### Treatment

**Figure 1:**
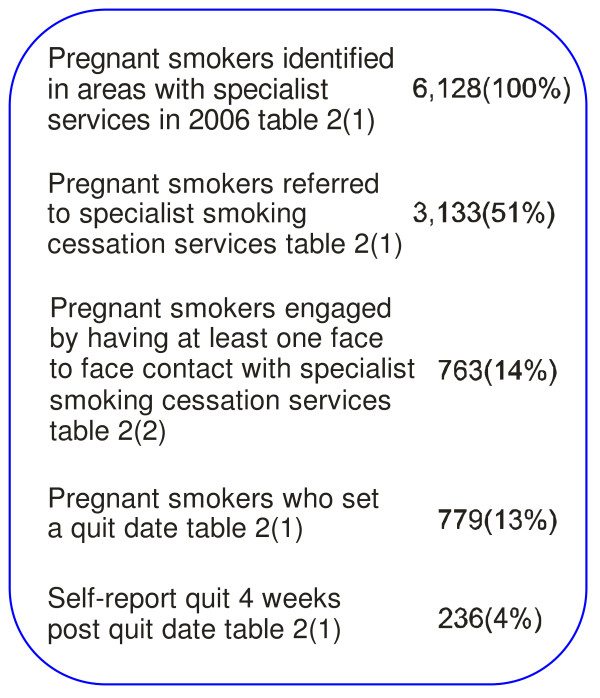
**Flow diagram of smokers from identification to self-report quit**. 1. Dumfries & Galloway, Fife, Greater Glasgow & Clyde, St John's Howden. Total identified smokers 6128. 2. Dumfries & Galloway, Fife, Greater Glasgow (breathe), St John's Howden. Total identified smokers 5306.

Treatment of those women who set a quit date is fairly universal throughout Scotland and entails using behavioural support usually with the help of Nicotine Replacement Therapy (NRT) to help overcome nicotine withdrawal. All specialist services in Scotland reported that women who quit used NRT [8]. This combination of support has been shown to be four times more effective than unassisted cessation [16]. Specialist home-based 119/409(29%), clinic-based 117/370(32%) and generic services 24/61(35%) all achieved comparable quit rates for those who set a quit date. These proportions may be amenable to some improvement as, in comparison, 40% quit rates for pregnant women have been reported by stop smoking services in England [17]. Improvements could be achieved by, for instance, better provision of NRT or improved training for specialist smoking cessation providers. Direct dispensing or prescription may increase the utility of NRT [18]. However, to achieve national targets in Scotland we need to more than double the number of pregnant smokers who quit. This cannot be achieved by merely improving quit rates for those who set a quit date. Major improvements in reach are needed so that more women access cessation services and set a quit date during pregnancy (figure 1).

### Identifying pregnant smokers

Few previous studies have had the data available to provide appropriate denominator estimates of current self-reported smokers within the population being treated. Most services use the number of smokers who engage as a proxy denominator and measure their success as the proportion of these clients who quit [18]. This approach takes no account of how hard the service tries to reach smokers. It is in fact a disincentive to try to reach difficult to engage groups who are thought to have limited success and will therefore reduce the proportion of clients who quit. The proportion of pregnant smokers identified at maternity booking is the starting point and needs to be more accurate if we are to improve reach. In most maternity hospitals nearly all women are asked by their routine midwife about their smoking status. There were exceptions, notably in two hospitals where 36% and 32% respectively of SMR02 maternity records had smoking status recorded as 'not known'. We are aware from previous work [6] that in one of these hospitals these missing data are, at least in part, a reflection of women not being asked about smoking often because midwives worry that the question will cause a rift with the patient that will affect their relationship throughout pregnancy or that other issues such as domestic violence are more important[6]. This problem has been overcome at the Southern General Hospital in Glasgow by taking some of the responsibility for identifying smokers away from busy routine midwifery staff and giving the responsibility to auxiliary staff who ask all pregnant women for a carbon monoxide breath test sample. The latter helps to overcome under-reporting of current smoking by women at booking. This system has allowed 87% of self-reported smokers to be referred (notified to specialist smoking cessation services) at the Southern General compared with just 39% at another Glasgow hospital where busy midwives are expected to ask for a carbon monoxide breath test using a similar opt-out system.

Another way to circumvent the difficulties of busy midwifery booking, under-reporting of smoking by pregnant women and the time and effort of referral would be to routinely test all maternity booking blood samples for cotinine, a nicotine metabolite [11]. All women with a positive test would be notified to specialist services and minimal intervention would be provided by specialist smoking cessation practitioners as a first telephone contact. Only once contact was made would the client be able to 'opt out' of the program. This would allow all pregnant smokers to be offered specialist support to help them stop smoking during pregnancy.

### Referring pregnant smokers

From table 2, 87% of self-reported smokers were identified and referred at the Southern General Hospital (SGH) compared with only 39% at the Princess Royal Maternity Hospital (PRMH). Both hospitals had the same opt-out clinic based service provision in the same city. We suggest the success at the SGH was due to auxiliary staff being responsible for carbon monoxide testing and referral of all smokers. Auxiliary nurses were not used in this way at the PRMH, the services were otherwise the same. More interesting is that similar proportions of **referred **smokers engaged at a clinic visit (SGH 106/573 - 18%, PRMH 146/703 - 21%), set a quit date (SGH 93/573 - 16%, 145/703 - 21%) and had quit smoking 4 weeks later (SGH 33/573 - 6%, PRMH 50/703 - 7%) in each of these hospitals. Smoking cessation targets are set in Scotland using **identified **smokers as the denominator. By referring nearly all identified smokers - 87%, SGH achieved a quit rate of 33/664 - 5% compared with PRMH which referred only 39% and achieved a quit rate of less than 3% - 50/1804. There is an extra cost of referring all pregnant smokers as many will not accept support as they are not ready to quit smoking. However, by referring all, the proportion who quit almost doubled as above. Unless the cost of providing such services is twice as much, which it is not, then referring all smokers and utilizing an opt-out policy at the time of initial telephone contact by specialist smoking cessation services will result in a lower cost per quitter.

### Initial contact by specialist smoking cessation services

Even if details of all smokers are given to specialist services, many smokers cannot be contacted. In Glasgow 38% of referrals were not useful because contact could not be made with the client [8,15]. The opt-out system could be improved substantially by making sure that multiple telephone contact details are gathered. Other ways to improve reach and engagement should also be explored and evaluated. One potentially promising innovation is the use of financial incentives to encourage women to use services, which is supported by accumulating evidence of effectiveness from four randomised controlled trials in the US including over 1200 patients [19].

### Engagement

Engagement - at least one face to face encounter with a specialist smoking cessation practitioner - was greater in areas using home-based support, where 50% of referred smokers engaged with services compared to only 20% with clinic-based support (Glasgow). The proportion engaged who set a quit date was lower in home-based areas 65%, nearly all clients (96%) who attended clinic-based support set a quit date. Taking this into account home-based support would increase quit rates, but at a greatly increased cost. One hundred and nineteen quits were recorded in home-based areas employing 5.3 specialist smoking cessation practitioners compared to 117 quits with 1.5 practitioners in the clinic-based service in Glasgow.

### Health inequalities

Routine data collection also allowed us to look at evidence for widening of health inequalities by providing smoking cessation services for pregnant women. Others have suggested that only affluent pregnant smokers will take up the offer of help and quit smoking. Table 3 indicates that this is not the case for Glasgow specialist smoking cessation services. Carstairs deprivation category describes material deprivation linked to major health indices [14]. In Glasgow most of the women who quit smoking lived in the most materially deprived areas 6&7 [8,15]. Indeed because smoking is so prevalent in deprived groups and fairly rare in affluent groups, it is difficult to see how a service that increases smoking cessation and reaches the most deprived groups (Table 3) can do anything but reduce overall health inequalities, as other studies of NHS stop smoking services have found [20].

This study suggests that routinely collected data documenting self-reported current smoking at maternity booking provides a reasonably accurate measure to use as a denominator for the number of current pregnant smokers in Scotland. If we accept this denominator then National Health Service funding should follow the need as shown by this denominator. Staffing arrangements identified in our study illustrate that resources are not currently distributed equitably with regard to need. Lothian Health Board area for example had 2.8 whole time equivalent specialist smoking cessation midwives for every 1000 self reported smokers (Table 2) whereas Greater Glasgow & Clyde had 1/1000. This inequality of service provision is not necessarily a reflection of unequal central funding, as local Health Boards decide how centrally allocated funds are spent.

## Conclusions

Smoking cessation services have traditionally been judged on the effectiveness of the intervention once the client has accepted treatment. However, for pregnant women and their unborn babies the issue of reach, particularly for materially deprived groups, is of equal concern. Gathering information that allows the denominator (number of pregnant smokers within a management area) to be ascertained provides services with a valid starting point for judging performance. Collecting information on referrals received and engagement achieved allows an assessment of the extent of reach and the staffing levels required. This type of information then needs to be considered alongside outcome data on the number of women who set a quit date and who quit smoking, ideally with biochemical validation. Policy makers and service providers need to move towards assessing this pathway of indicators, starting with the denominator 'current pregnant smokers', if sensible decisions regarding service development, resource allocation and target setting to reduce smoking in pregnancy are to be made in the future.

## Abbreviations

ISD: Information Services Division; NHS: National Services Scotland; MDS: National Minimum Dataset; SMR02: Standard Morbidity Record 02; StJ: St John's Hospital West Lothian; PRMH: Princess Royal Maternity Hospital Glasgow.

## Competing interests

The authors declare that they have no competing interests.

## Authors' contributions

DMT wrote the manuscript, helped analyse the data, and helped design the study; SM, DE and LB helped design the study, collected questionnaire data from each health board and individual smoking cessation services and assisted with drafting and editing the manuscript; DS accessed and analysed routinely collected data from the Information Services Division, Scottish Government and helped with drafts of the manuscript; LG provided information from the National Minimum Dataset (MDS) for smoking cessation services - Information Services Division, NHS National Services Scotland, and helped draft the manuscript. DMT is the guarantor of the work. All authors have read and approve the final manuscript.

## Authors' information

Professor Linda Bauld, University of Bath is a member of the UK Centre for Tobacco Control Studies (UKCTCS) which is a consortium of academics from 9 universities conducting research on tobacco control and smoking cessation. Along with Dr Tim Coleman, she has lead role for smoking cessation during pregnancy in UKCTCS. Douglas Eadie and Susan MacAskill are senior researchers at the Institute for Social Marketing University of Stirling and Open University, part of the UK Centre for Tobacco Control Studies. They led the NHS Health Scotland funded audit of smoking cessation in pregnancy services in Scotland with Professor Bauld. Dr David Tappin director of the Paediatric Epidemiology and Community Health (PEACH) Unit, an academic unit of Glasgow University that seeks to link Public Health and Child Health research. He supervised Dr Shipton a postdoctoral statistician in her work for this paper while she was attached to the PEACH unit and the Information Services Division (ISD), NHS National Services Scotland. Dr Tappin has a special interest in smoking cessation during pregnancy. Linsey Galbraith is Principal Information & Development Officer Information Services Division (ISD), NHS National Services Scotland. She is responsible for monitoring of the Minimum Dataset (MDS) collected in Scotland to document all individuals attending smoking cessation services who have set a quit date and also those who have successfully quit 4 weeks, 3 months and 12 months later.
